# Prognostic Value of Echocardiographic RV–PA Coupling in Advanced Heart Failure

**DOI:** 10.1111/echo.70348

**Published:** 2025-11-18

**Authors:** Tadafumi Sugimoto

**Affiliations:** ^1^ Department of Cardiology Nagoya City University Mirai Kousei Hospital Nagoya Japan

1

The evolving stages of heart failure are associated with right ventricular to pulmonary arterial (RV‐PA) uncoupling, impaired gas exchange, and ventilatory inefficiency during exercise [1]. The ratio of tricuspid annular plane systolic excursion (TAPSE) to pulmonary artery systolic pressure (PASP) has been proposed as a simplified surrogate of RV to PA coupling, reflecting the compensatory adaptation of contractility to increased afterload. In this issue of the *Echocardiography*, Tanyeri et al. report that among noninvasive parameters reflecting RV–PA coupling, TAPSE/PASP, fractional area change (FAC)/PASP, and RV ejection fraction (RVEF)/PASP demonstrated significant associations with adverse outcomes in patients with advanced heart failure, indicating that these indices may serve as valuable markers for the early identification of high‐risk individuals [2]. The study cohort had a mean age of 54 years, with a mean left ventricular (LV) end‐diastolic diameter of 68 mm, a mean LVEF of 21%, a mean TAPSE of 16.4 mm, and a mean PASP of 40.8 mmHg [2]. In previous studies evaluating the clinical significance of RV–PA coupling in patients with non‐ischemic DCM and advanced heart failure, TAPSE/PASP did not predict a composite endpoint of LV assist device implantation and all‐cause mortality within 1 year [3]. Interestingly, in the study by Tanyeri et al., TAPSE/PASP was shown to predict a composite of LV assist device implantation, heart transplantation, and all‐cause mortality over a median follow‐up of 452 days. In the study by Ishiwata J et al., the cohort had a mean age of 44 years, a mean LV end‐diastolic diameter of 71 mm, a mean LVEF of 22%, TAPSE of 15.3 mm, and PASP of 31.8 mmHg (estimated using the Hozo method). Compared with Tanyeri et al., the larger LV size and lower TAPSE and PASP suggest that Ishiwata et al. included patients with more advanced heart failure. These findings imply that in advanced stages, when PASP declines due to impaired RV contractility limiting the tricuspid pressure gradient, the utility of TAPSE/PASP as an RV–PA coupling index may be limited, whereas indices such as FAC/PASP or RV free‐wall strain (RVFWS)/PASP may provide a more reliable assessment.

The development of heart failure, regardless of LVEF, essentially begins with an elevation in LV end‐diastolic pressure, which is subsequently followed by an increase in left atrial pressure [4], the left ventricle's upstream chamber. Since there is no valve between the left atrium and the pulmonary veins, elevated left atrial pressure leads to increased pulmonary venous pressure. This, in turn, raises pulmonary capillary pressure and impairs gas exchange in the alveoli [5]. When pulmonary venous pressure becomes markedly elevated, a compensatory mechanism is activated to limit further increases in pulmonary capillary pressure by increasing pulmonary vascular resistance. However, this compensation results in elevated pulmonary artery pressure, thereby increasing right ventricular afterload. In the early stages of right heart loading, RV–PA coupling is preserved. Yet, as right ventricular afterload continues to rise and compensatory mechanisms become insufficient, right ventricular contractile indices decline relative to PASP, leading to progressive RV–PA uncoupling.

Echocardiographic indices of RV–PA coupling, including TAPSE/PASP, FAC/PASP, and RVEF/PASP, are valuable for diagnosing heart failure, assessing disease severity, and predicting prognosis. Nevertheless, their evaluation at rest has inherent limitations [6]. Assessing hemodynamic responses during exercise can provide additional insight, even in early‐stage heart failure, enhancing the accuracy of diagnosis, pathophysiological assessment, and prognostic stratification. Consistently, in the study by Tanyeri et al. [2], patients who experienced events exhibited lower peak oxygen uptake and greater exercise ventilatory inefficiency than those without events. In heart failure, as at rest, exercise also initiates with an elevation in LV end‐diastolic pressure, followed by increases in left atrial pressure and pulmonary artery pressure, along with a sequential decline in right ventricular contractile indices [7], with exercise‐induced mitral regurgitation contributing simultaneously [8]. These hemodynamic changes are closely associated with reductions in peak oxygen uptake and exercise ventilatory efficiency [9], highlighting the potential for further advances in this area.
